# Metabolic networks of plasma and joint fluid base on differential correlation

**DOI:** 10.1371/journal.pone.0247191

**Published:** 2021-02-22

**Authors:** Bingyong Xu, Hong Su, Ruya Wang, Yixiao Wang, Weidong Zhang

**Affiliations:** 1 School of Pharmaceutical Sciences, Jilin University, Changchun, Jilin, China; 2 Hangzhou Heze Pharmaceutical Technology CO.,LTD, Hangzhou, Zhejiang, China; 3 Department of Pharmacy and Examination, Daqing Medical College, Daqing, Heilongjiang, China; Weill Cornell Medical College in Qatar, QATAR

## Abstract

Whether osteoarthritis (OA) is a systemic metabolic disorder remains controversial. The aim of this study was to investigate the metabolic characteristics between plasma and knee joint fluid (JF) of patients with advanced OA using a differential correlation metabolic (DCM) networks approach. Plasma and JF were collected during the joint replacement surgery of patients with knee OA. The biological samples were pretreated with standard procedures for metabolite analysis. The metabolic profiling was conducted by means of liquid mass spectrometry coupled with a AbsoluteIDQ kit. A DCM network approach was adopted for analyzing the metabolomics data between the plasma and JF. The variation in the correlation of the pairwise metabolites was quantified across the plasma and JF samples, and networks analysis was used to characterize the difference in the correlations of the metabolites from the two sample types. Core metabolites that played an important role in the DCM networks were identified via topological analysis. One hundred advanced OA patients (50 men and 50 women) were included in this study, with an average age of 65.0 ± 7.6 years (65.6 ± 7.1 years for females and 64.4 ± 8.1 years for males) and a mean BMI of 32.6 ± 5.8 kg/m^2^ (33.4 ± 6.3 kg/m^2^ for females and 31.7 ± 5.3 kg/m^2^ for males). Age and BMI matched between the male and female groups. One hundred and forty-five nodes, 567 edges, and 131 nodes, 407 edges were found in the DCM networks (p < 0.05) of the female and male groups, respectively. Six metabolites in the female group and 5 metabolites in the male group were identified as key nodes in the network. There was a significant difference in the differential correlation metabolism networks of plasma and JF that may be related to local joint metabolism. Focusing on these key metabolites may help uncover the pathogenesis of knee OA. In addition, the differential metabolic correlation between plasma and JF mostly overlapped, indicating that these common correlations of pairwise metabolites may be a reflection of systemic characteristics of JF and that most significant correlation variations were just a result of "housekeeping” biological reactions.

## Introduction

Osteoarthritis (OA) is a common orthopedic disease with no effective treatment in clinic. **[1]**. A growing number of studies have suggested that OA is a systemic metabolism disorder associated with components of the metabolic syndrome. However, there are still some researchers who believe that OA should not considered a systemic disease, as it only affects the joints and not other organ systems **[2–4]**. Plasma primarily comes from the systemic circulation, while joint fluid (JF) is a viscous ultrafiltrate of plasma and also affected by its localized environment **[5]**. Given the close relationship between JF and blood and joint tissues, blood samples are usually used in OA research based on the assumption that the metabolic characteristics between plasma and JF are similar **[6]**. However, this assumption has yet to be fully tested, and comprehensive investigations are needed. In our previous work, we studied the relationship between plasma and joint fluid based on absolute metabolite concentrations, found a modest relationship **[7]**.

Differential correlation metabolic (DCM) network analysis is a powerful approach to the study of biological interaction when biosystems are undergoing differential changes based on the environment, tissue type, and disease state **[8]**. Compared to investigating the absolute concentrations of metabolites, DCM network analysis focuses on the interrelation between the whole metabolites affected by in vivo or in vitro influencing factors, such as genetic and environmental factors. Analyzing the reconstruction of metabolic networks affected by such various factors provides a unique perspective in studying the whole dynamic change of a biological system **[9, 10]**. In this paper, we adopted the DCM network approach to analyze the metabolic characteristics between plasma and JF. A differential analysis was used to quantify the correlation variations of pairwise metabolites between plasma and JF samples and to draw the overall interconnection structure of the differently related metabolites. The design of our research approach is different from previous studies in that we study the correlation variations of pairwise metabolites by comparing different biological systems, i.e., plasma and JF, to help elucidate whether OA is a systemic or localized metabolic disorder.

## Materials and methods

### Study subjects

Advanced OA patients in need of total knee replacement (TKR) surgery were recruited at the First Hospital of Jilin University (Changchun, China). All subjects understood the content and purpose of the study, and all participants signed an informed consent form. Demographic information, such as height, weight, age, and primary concomitant diseases, was collected during the preparation for the TKR surgery using a self-administered questionnaire that was then confirmed by the nurse. This study was approved by the Ethics Committee of Jilin University (No. 2020–308).

### Preparation of plasma and JF samples

Subjects fasted for more than eight hours before sampling. The plasma was separated from the whole blood according to the standard protocol. The JF was obtained by inserting syringes into the articular cavity of the knee and aspirating before TKR surgery. The collected samples were transferred to polyethylene tubes and placed in a liquid nitrogen tank for subsequent analysis. The samples were processed and analyzed according to the manual included in the AbsoluteIDQ p180 assay kit of Biocrates (Biocrates life sciences ag, Austria).

### Metabolic profiling

The metabolic profiling of the plasma and JF samples was conducted using Water’s triple quadruple liquid mass spectrometer combined with the Biocrates AbsoluteIDQ p180 assay kit. This powerful method can measure 186 metabolites (including 90 glycerophospholipids, 40 acylcarnitines, 21 amino acids, 19 biogenic amines, 15 sphingolipids, and one hexose). More information on these metabolites is presented in **S1 Table**. Over 90% the metabolites in each sample were successfully determined.

The metabolite concentrations obtained from the AbsoluteIDQ kit were used for the analyses. For further analysis, we selected the metabolites that could be measured in more than 80% of the total samples **[11]**, and half of the minimum value was used to replace the blank values of the metabolites **[12]**.

### Statistical methods

The DCM network analysis approach was previously developed by our research team and was applied to calculate the correlation values of pairwise metabolites in different phenotypic region groups, a process followed by subtracting the correlation values and constructing the differential network by the significantly different pairwise metabolites **[13]**. In the study, this approach was used to construct a global network to present the differentially related pairwise metabolites for plasma and JF. Differences between the two correlations could be tested using the following Eqs (1)–(3):

$$r_{diff}\left(i,j\right)=\sqrt{\frac{n_{plasma}-3}{2}}\times z_{plasma}\left(i,j\right)-\sqrt{\frac{n_{SF}-3}{2}}\times z_{JF}\left(i,j\right)$$ (1)

$$z_{plasma}\left(i,j\right)=\frac{1}{2}ln[\frac{1+r_{plasma}(i,j)}{1-r_{plasma}(i,j)}]$$ (2)

$$z_{JF}\left(i,j\right)=\frac{1}{2}ln[\frac{1+r_{SF}(i,j)}{1-r_{SF}(i,j)}]$$ (3)

Where i and j are metabolites, n_plasma_ and n_JF_ denote the total number of the plasma and JF samples, respectively, r_diff(i, j)_ denotes the differential correlations of i and j, and *z* is the Fisher’s z-transformation of correlation coefficient *r*. Specifically, r_diff_ describes the change in the correlations and was calculated by subtracting the correlation of the JF samples from that of the plasma samples **[14–16]**. Permutation testing was adopted to test the significant differential correlations. The network visualization was generated by the software Cytoscape 3.0.

In networks, the node is the most important parameter and is measured by its nod degree and centrality, including betweenness centrality and closeness centrality. Highly connected nodes usually show a high degree, high betweenness, and closeness centrality and are regarded as “network hubs”, indicating their important role in the network.

## Results

### Demographics and anthropometrics

One hundred OA patients (50 female and 50 male) who underwent TKR surgery were involved in this research. Blood and JF samples were collected from each participant. The mean age of the patients was 65.0 ± 7.6 years, for which the average age was 65.6 ± 7.1 and 64.4 ± 8.1 years among the female and male groups, respectively. The mean BMI was 33.4 ± 6.3 kg/m^2^ for the females, 31.7 ± 5.3 kg/m^2^ for the males, and 32.6 ± 5.8 kg/m^2^ for both sexes combined. Both age and BMI had no significant difference between the male and female participants.

### Metabolite correlations in plasma and JF samples

**Fig 1:**
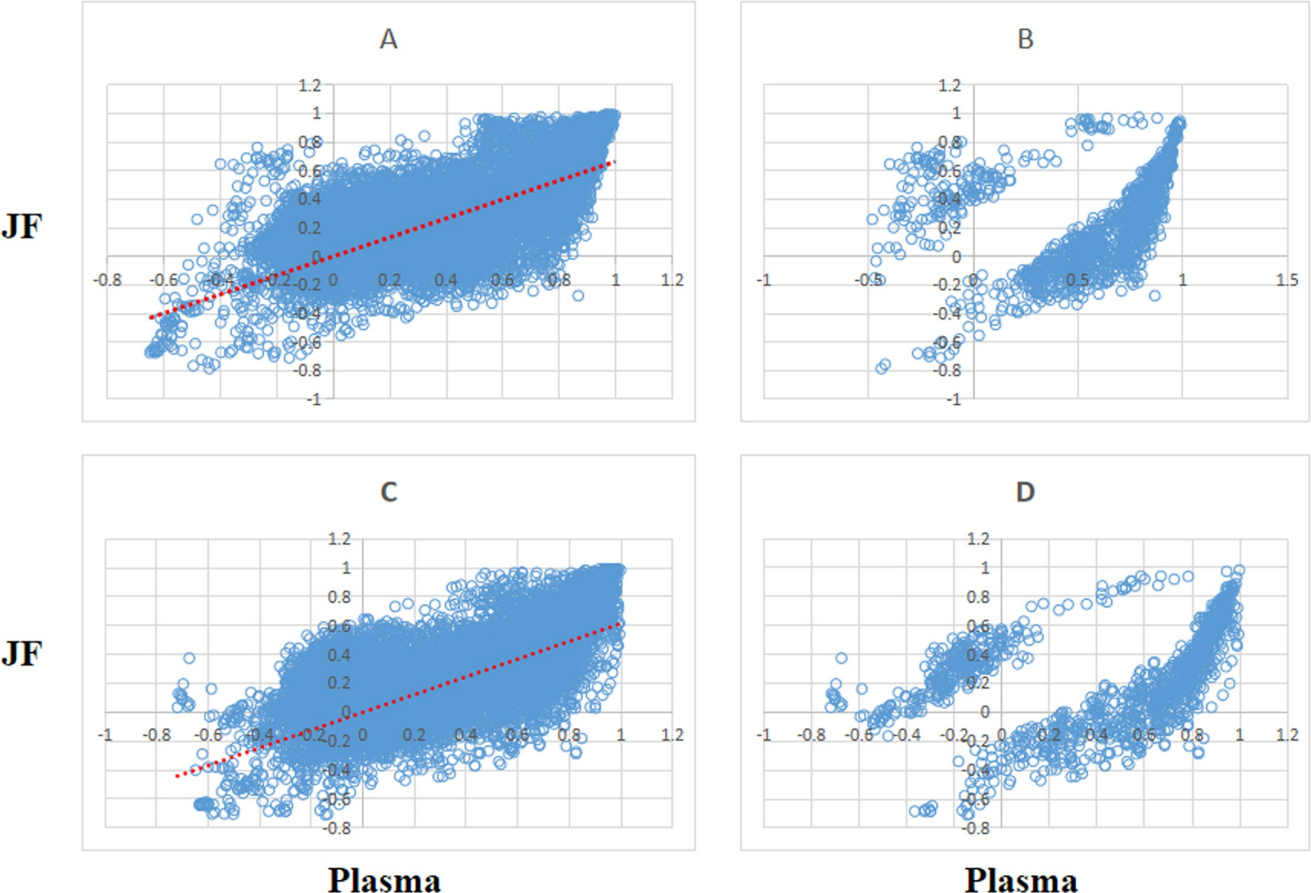
(A, C) Scatter of metabolite pair correlations in plasma (x axis) and JF (y axis) and (B, D) identification of significant (p-value cutoff 0.05 using a 1000-fold permutation test) pairs. (A and B for female, C and D for male).

The metabolite correlations of the 167 metabolites were calculated using Pearson’s correlations method. P value < 0.05 were used to select the significant pairwise correlations. After the calculation and selection, the majority of the 13,861 pairs showed positively correlations in both the plasma and JF. The scatter plot of all the pairwise metabolites as well as their correlations is shown in **Fig 1**, in which the majority of the significant correlations of pairwise metabolites in the plasma and SF are the same for both men and women. The high overlap of the pairwise metabolite correlations between the plasma and SF suggested that they share common metabolic characteristics.

#### Differentially correlated metabolites

There were a total of 1331 pairs of metabolites in the female group showing significant differential correlations (p < 0.05), of which 1,145 pairs was positive and 186 pairs was negative. After multiple testing, 18 pairs of metabolites with positive differential correlations were still found to be significant (p < 3.6×10^−6^, 0.05/13861), with the most significant pair of metabolites being Kynurenine and PCaaC24:0 (r-diff = 7.85). There were a total of 1140 pairs of metabolites showing significant differential correlations in the male group (p < 0.05), of which 841 pairs was positive and 299 pairs was negative. After multiple testing, 21 pairs of metabolites (16 positive differential correlations and 5 negative differential correlations) were still significant (p < 3.6×10^−6^). The strongest pair of metabolites were C14:2-OH and C16:2 (r-diff = 9.78) and Creatinine and PCaeC42:0 (r-diff = -4.89), respectively.

### Differential correlation metabolite network

**Fig 2:**
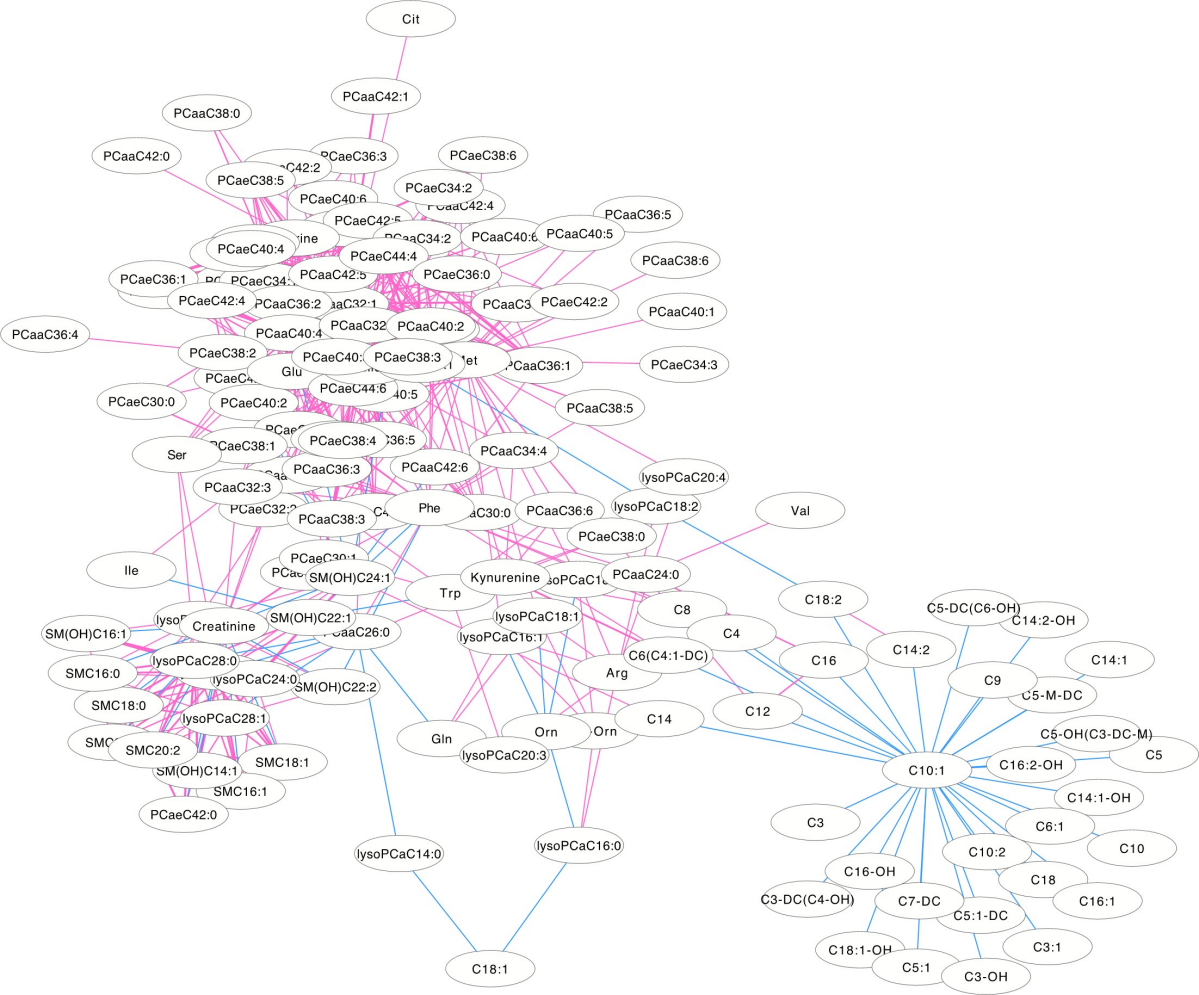
The differential correlation network by comparing the female plasma and JF data. Only pairs of metabolites that have significant differential correlations (P<0.01) in both datasets are shown.

**Fig 3:**
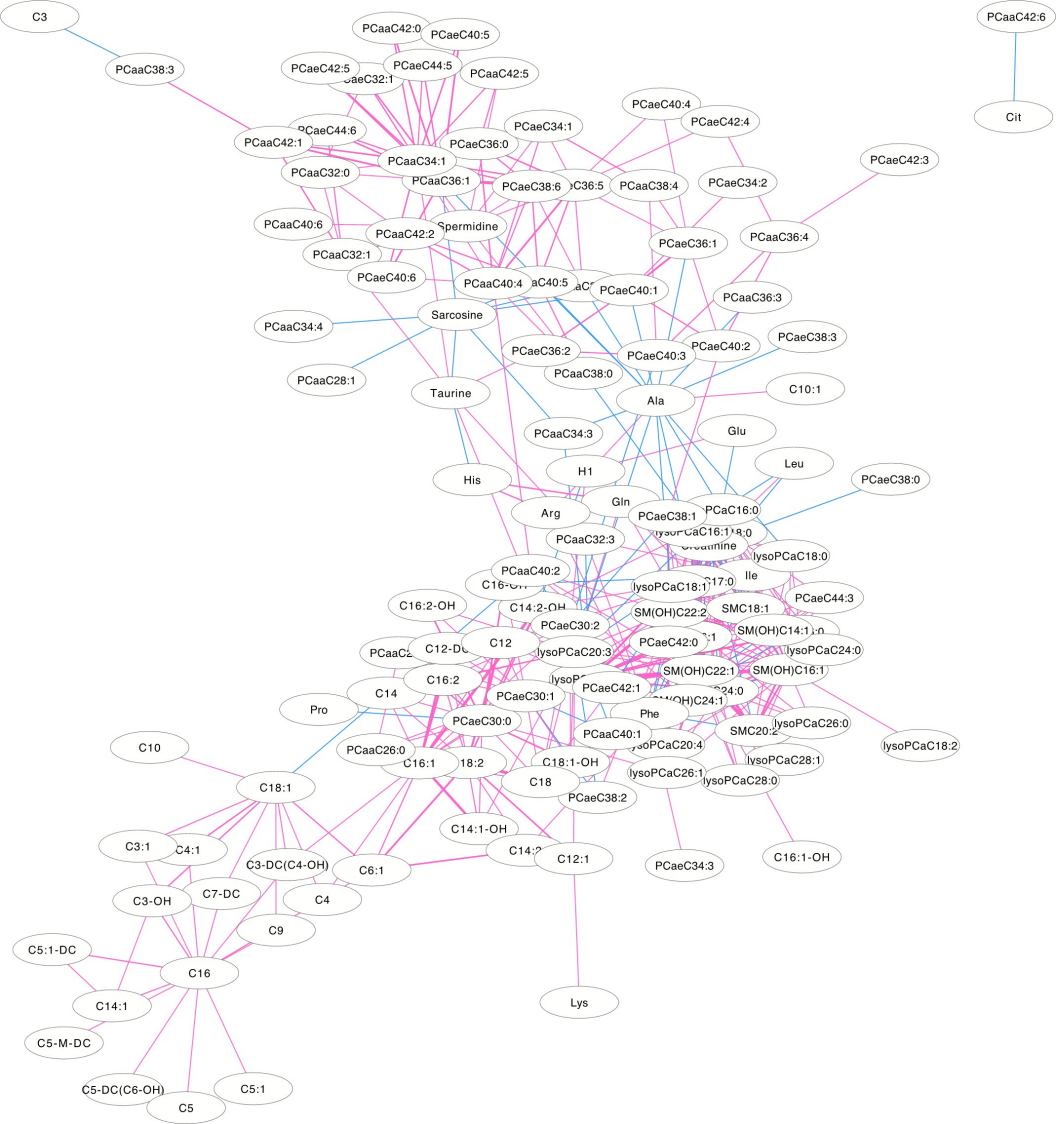
The differential correlation network by comparing the male plasma and JF data. Only pairs of metabolites that have significant differential correlations (P<0.01) in both datasets are shown.

The differentially correlated metabolites were used to build the differential correlation network. All the positive differential correlated pairwise metabolites are denoted with red lines, while negative correlations are denoted in blue (**Figs 2 and 3**). The parameters of nod degree, betweenness centrality and closeness centrality extracted from the DCM networks (p<0.05) were shown in **S1 and S2 Tables**, respectively. In the female groups, there is a mean value of 7.82 for the node degree, 0.32 for closeness centrality and 0.02 for betweenness centrality in the network, in which PC aa C32:0 was the core metabolite with a degree of 42, indicating a robust information flow and connectivity in the network. In the male groups, most of the metabolite pairs had positive differential correlations. The mean value of 6.21 was the node degree, 0.29 for closeness centrality and 0.02 in the network, in which SM(OH)C22:1 was the core metabolite with a degree of 24.

### Identification of core metabolites in the network

**Fig 4:**
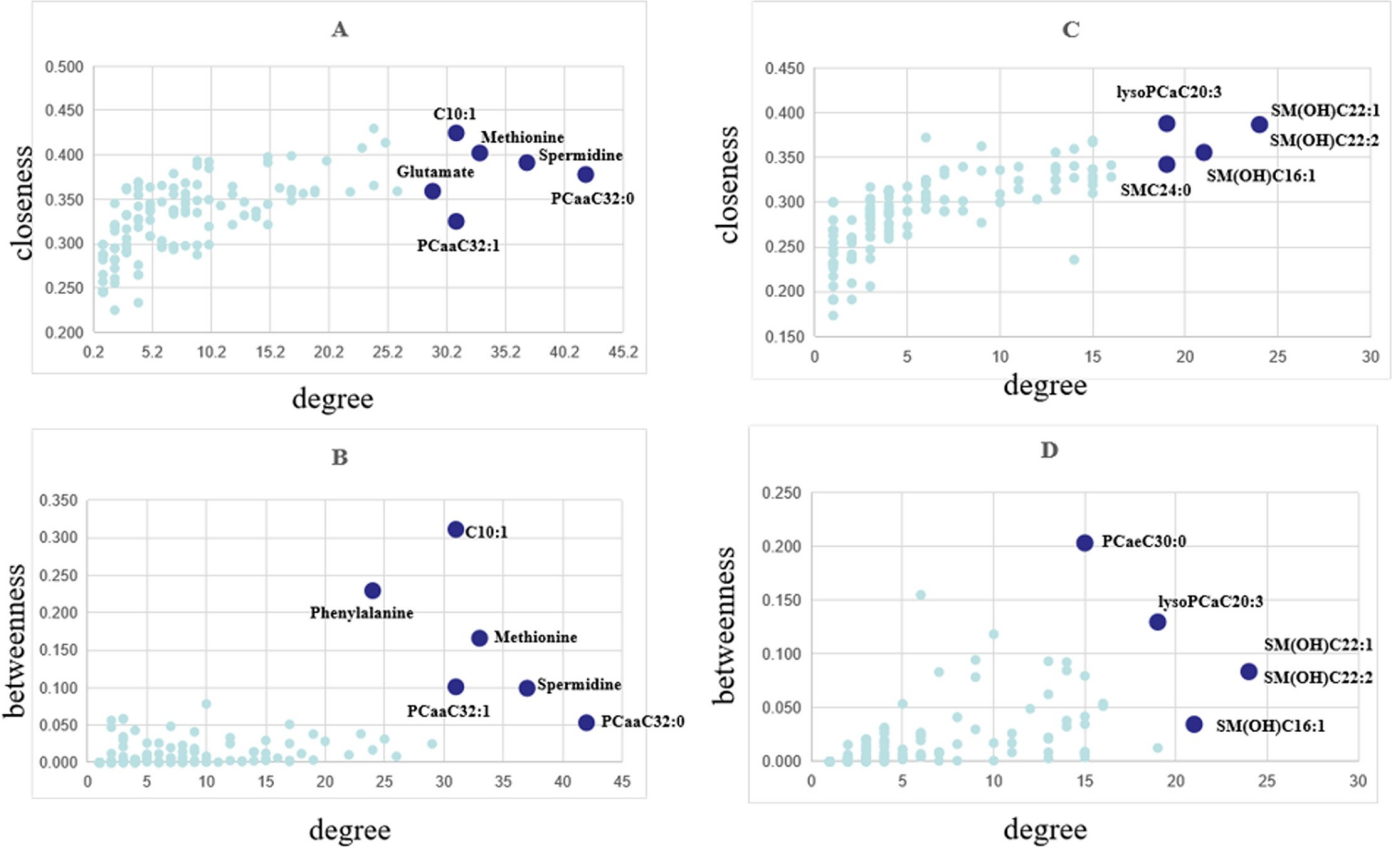
Node importance characterized by (A, C) closeness centrality and (B, D) betweenness centrality in relation to node degree. (A and B for female, C and D for male).

In the DCM networks (p<0.05), the core metabolites playing a key role, usually as hubs or bottlenecks, were identified. **Fig 4** shows the core metabolites based on the high degee, betweenness and closeness centralities. Six core metabolites were identified in female group as key nodes, in which 5 metabolites were common in both centralities. In male group, 5 core metabolites were identified as key nodes, in which 4 metabolites were common in both cetralities. The significant metabolites differentially expressed between JF and plasma and males and females were shown in **S4 Table**.

**Fig 5:**
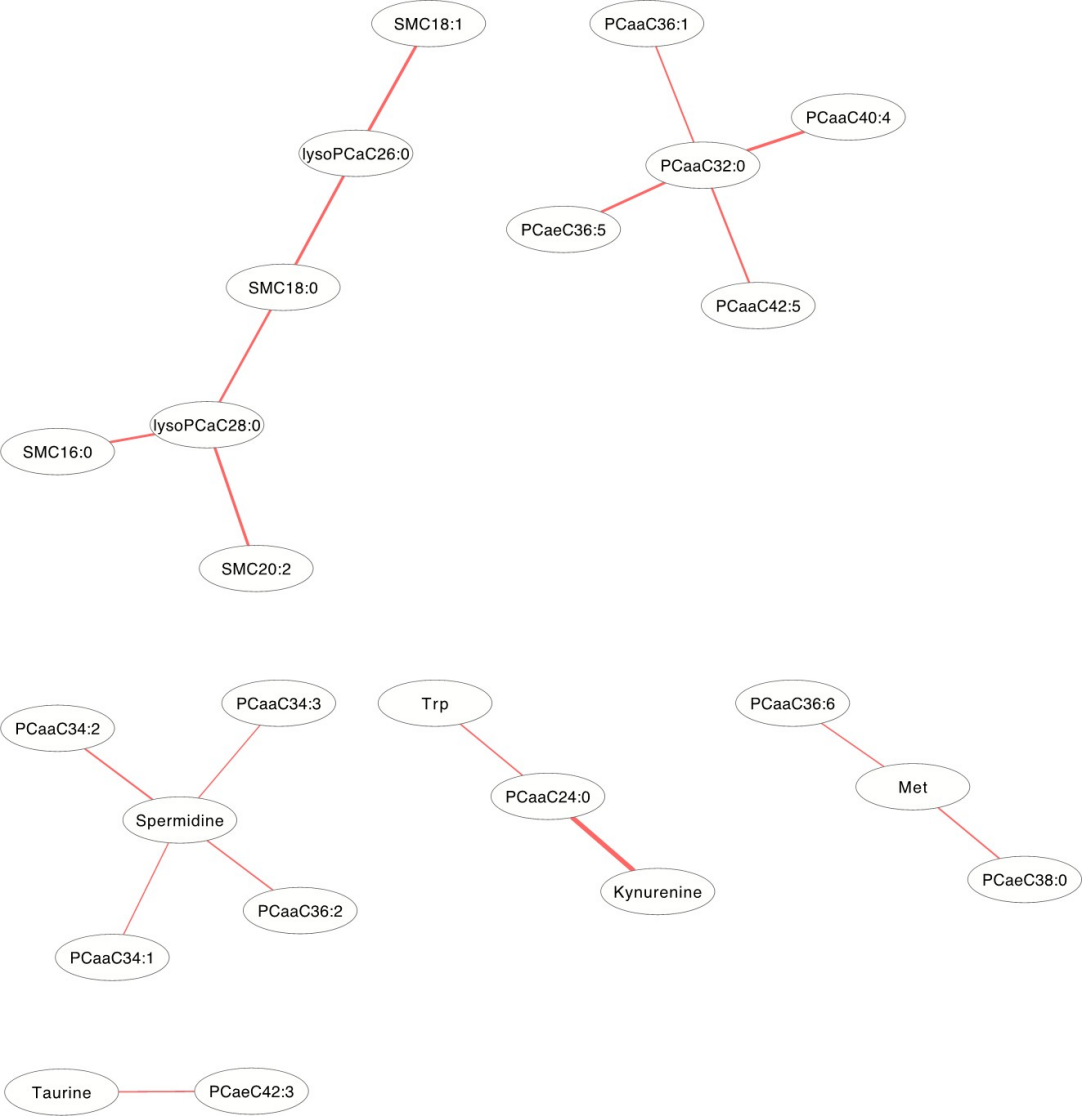
The differential correlation network by comparing the female plasma and JF data. Only pairs of metabolites that have significant differential correlations (P<3.6×10^−6^) in both datasets are shown.

**Fig 6:**
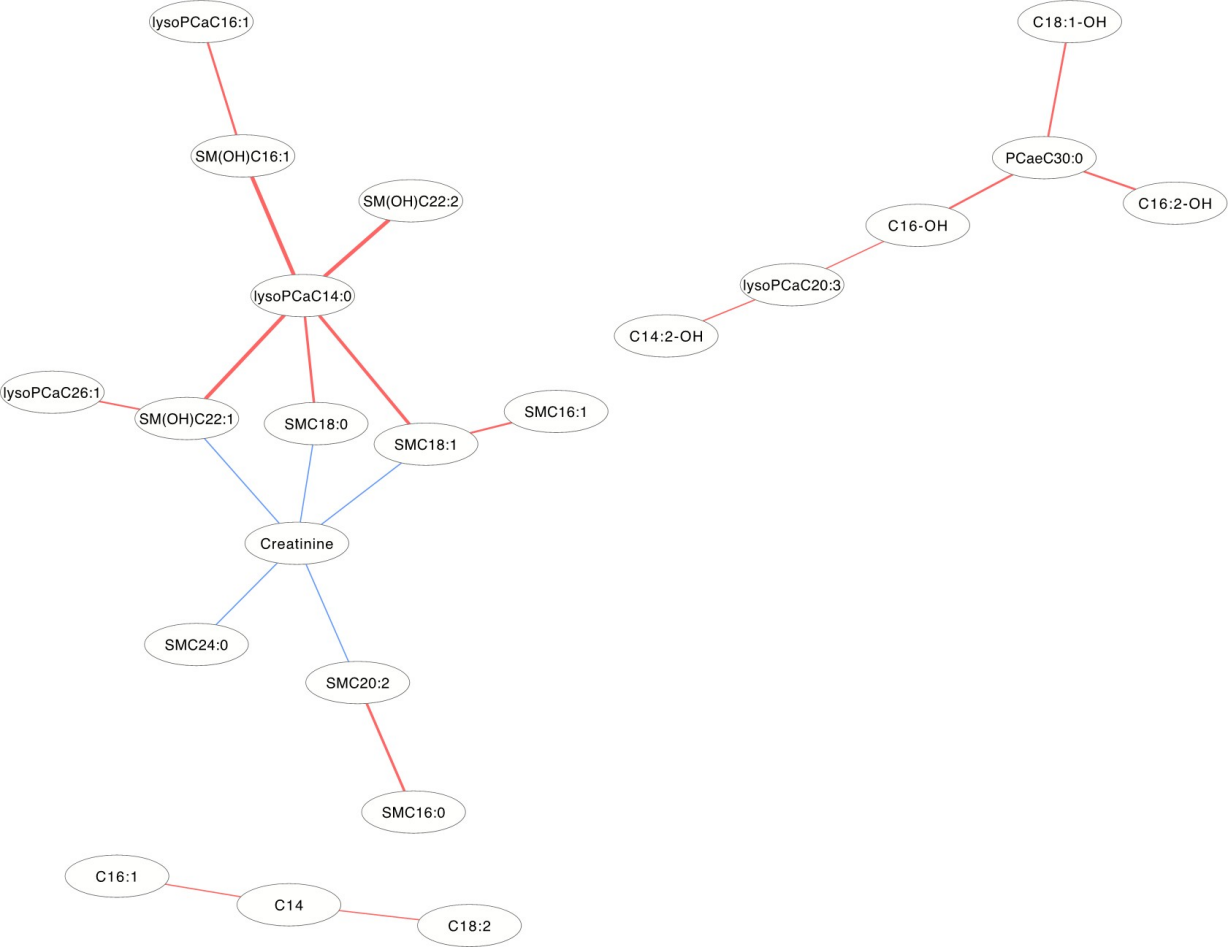
The differential correlation network by comparing the male plasma and JF data. Only pairs of metabolites that have significant differential correlations (P<3.6×10^−6^) in both datasets are shown.

In the DCM networks (p<3.6×10^−6^) of **Figs 5 and 6**, it is easy to see that 3 metabolites (PCaaC32:0, Spermidine and lysoPCaC28:0) in female and 5 metabolites (Creatinine, lysoPCaaC14:0, SM(OH)C22:1, SMC18:1 and PCaaC30:0) in male were identified as core metabolites in the networks.

## Discussion

In general, blood has always been a substitute for joint fluid based on the understanding that joint fluid is a filtrate of plasma with similar metabolic characteristics. However, this view has not been fully studied. In this paper, we studied the metabolic differences of plasma and joint fluid using a metabolomics method. DCM network analysis was adopted to determine differential metabolism characteristics between plasma and JF. As far as I know, this study is the first to use differential network analysis to study the metabolic differences between plasma and JF.

DCM networks were used to describe the global inter-connected structure of the pairwise metabolites that identified significant correlation variations between the plasma and JF samples. Some key metabolites were identified as controlling the connectivity and information flow of the differential network. A high-degree overlap of the correlated metabolite pairs was found when the metabolite correlations were analyzed separately in the plasma and JF samples **(Fig 1)**, which indicated that most of the metabolite correlations were common between the plasma and JF samples. This is consistent with the fact that plasma is the main source of joint fluid. However, there were still some differentially correlated metabolites identified in the analysis, which suggested different underlying biological processes between the JF and plasma samples, and these metabolites may be related to a joint-specific metabolism. In the DCM network, several more positive differential correlations were observed than negative ones (**Figs 2 and 3**), which indicated that some biological processes might have been active in the plasma and compromised in the JF samples. However, there were still some differential correlations that increased in the JF. For example, the correlations of acylcarnitines were more active in the JF than the plasma in female group.

It is thought that women and men have different abilities to cope with illnesses **[17, 18]**. The incidence and clinical symptoms of knee OA differ markedly between men and women. For example, old women have a higher incidence of OA and usually have a more obvious pain response during the onset of OA compared with men **[19, 20]**. Although the mechanisms that lead to the differences in the incidence and severity of OA between men and women are still unknown, metabolic differences and molecular events may play a part. In our previous investigations, a large number of metabolites were found to be showing gender-specific associations **[21, 22]**. In the female group, the metabolites with positive differential correlations mainly came from the class glycerophospholipids, while the negative differential correlations mainly came from acylcarnitines. In the male network, most of the differential correlations were positive, and sphingolipids were the main metabolites that contributed to the differential correlation between the JF and plasma samples.

We performed a 1000-fold permutation testing and the significance level of the differential correlation of each metabolite pair was assessed separately, mostly due to the purpose of analyzing many specific differential correlations. We also did the Bonferroni multiple test for the correlations to get the true level. The number of significant differential correlated pairs of metabolites became very small, but the main key nodes (core metabolites) were still similar. The difference may attribute to the Bonferroni approximation is too conservative. Although the nominal significance level could be different from its true level, but as some preliminary findings of the research, this could be adequate.

Glycerophospholipids are the major components of cellular membranes, and play a role in signal transduction, the regulation of membrane transport, and so on **[23, 24]**. Many studies have shown that the composition and concentrations of glycerophosphatides in the JF samples of OA patients have disturbed. Cartilage tissue is coated with a thin layer of surfactant phospholipids to promote lubrication **[25]** and load-bearing **[26]**. These phospholipids were mainly unsaturated phosphatidylcholines, which not only have anti-friction properties **[27]** but also form important components and maintain the necessary metabolic and physiological functions of joints. Therefore, they may have a potential effect on the protection and treatment of OA **[28]**. Zhai et al. studied 139 knee OA patients from a 24-month clinical trial cohort, and revealed an association between the serum ratio of lysophosphatidylcholine 18:2 (lysoPC 18:2) to phosphatidylcholine 44:3 (PC44:3) and the cartilage volume loss in the lateral compartment and with joint degradation markers, COMP and MMP1**[29]**. In our previous studies, we identified the ratio of lysoPCs to PCs as a novel metabolic marker for predicting advanced knee OA, and also demonstrated that altered phosphatidylcholine metabolism was associated with both OA and diabetes mellitus **[21, 30]**.

Carnitine and its acyl esters, acylcarnitines, are an important class of metabolites and play an important role in many biological processes, such as the β-oxidation metabolism of fatty acids. Acylcarnitines can be found in a wide variety of species due to the presence of different lengths of acyl chains and isomers **[31]**. L-carnitine is widely present in the body and is particularly abundant in mitochondria. The adrenal gland has been found to have the highest concentrations of L-carnitine, followed by the heart, bone, muscle, adipose tissue, and liver **[32]**. Carnitine has been reported to have the following effects: anti-aging, lowering blood cholesterol and triglycerides, weight loss. Moreover, it has been reported that acylcarnitines have the potential to activate inflammation **[33]**. Acylcarnitine disruption usually leads to obesity, type 2 diabetes, and cardiovascular diseases **[34, 35]**. Kaspar et al. compared the metabolic profile of lipid metabolism-related compounds and arterial stiffness in OA patients and in controls using the targeted metabolomic approach. They found decreased levels of acylcarnitines in OA patients. Furthermore, medium-and long-chain acylcarnitines associated independently with arterial stiffness and were related to radiographic severity of OA. Thus, acylcarnities might play an important role in the association between OA and cardiovascular diseases (CVD) **[36]**.

Sphingolipids are a fundamental class of molecules involved in structural of eukaryotic membranes **[36]**. Maintaining the metabolic balance of sphingolipids is of great significance to the normal physiological activities of cells. It has been proven to have the ability of regulating cell proliferation, apoptosis, and inflammation and also plays an important role in the pathogenesis of OA and RA **[37–40]**. Kosinska found that the JF of 19 SM species was 2.4 times higher in the early stage and 4.8 times higher in the late stage of OA than in the control group **[41]**. Moreover, sphingomyelins were positively correlated with the severity of osteoarthritis in mouse and canine OA models **[42, 43]**. Sphingolipid metabolism disruption has also been associated with cartilage damage in OA models **[44]**. Kosinska et al. made a lipidomic analysis to compare intra-individually a broad spectrum of phospholipid and sphingolipid species found in serum and SF of the same patients. They revealed that human serum often reflect the alterations in lipid species levels seen in SF of OA patients as compared to normal controls **[45]**.

## Conclusion

This study suggested that OA may be related to local joint metabolism and is not exactly equal to a systemic metabolic disease, as a significant difference in a part of correlations of metabolite pairwise was found between plasma and JF based on DCM networks analysis. As far as I know, this is the first study using a DCM network approach to analyze the metabolic differences between plasma and JF. The core metabolites that play an important role in the network were identified and deemed sex-specific. These differential metabolites may be closely related to the characteristic metabolism of joint tissue. Further study of these metabolites will help reveal the pathogenesis of osteoarthritis. In addition, in established models of differential metabolic correlations, most of the metabolic profiles of plasma and joint fluid overlap. This suggests that plasma and joint fluid share some common systemic metabolic characteristics.

## Supporting information

S1 Table List of metabolite concentrations determined using the Biocrates AbsoluteIDQ kit. (DOCX)

S2 Table Node importance in female group. (XLSX)

S3 Table Node importance in male group. (XLSX)

S4 Table Significant metabolites differentially expressed between JF and plasma in males and females. (DOC)
