# Supplementary material for: Metabolic networks of plasma and joint fluid base on differential correlation
Source: PLoS One. 2021 Feb 22;16(2):e0247191. doi: 10.1371/journal.pone.0247191 (PMC7899361; doi:10.1371/journal.pone.0247191)
Supplement: S4 Table — (DOC) [file pone.0247191.s004.doc]

**S4 Table.** Significant metabolites differentially expressed between JF and plasma in males and females

| **Gender** | **Name** | **Degree** | **Closeness** | **Betweenness** |
| --- | --- | --- | --- | --- |
| F | **PCaaC32:0** | 42 | 0.378 | 0.053 |
| F | **Spermidine** | 37 | 0.391 | 0.099 |
| F | **Methionine** | 33 | 0.402 | 0.166 |
| F | **PCaaC32:1** | 31 | 0.425 | 0.101 |
| F | **C10:1** | 31 | 0.325 | 0.311 |
| F | **Glutamate** | 29 | 0.359 | 0.025 |
| M | **SM(OH)C22:1** | 24 | 0.388 | 0.087 |
| M | **SM(OH)C22:2** | 24 | 0.387 | 0.084 |
| M | **SM(OH)C16:1** | 21 | 0.356 | 0.035 |
| M | **lysoPCaC20:3** | 19 | 0.388 | 0.130 |
| M | **SMC24:0** | 19 | 0.342 | 0.013 |
